# Web-based computer adaptive assessment of individual perceptions of job satisfaction for hospital workplace employees

**DOI:** 10.1186/1471-2288-11-47

**Published:** 2011-04-17

**Authors:** Tsair-Wei Chien, Wen-Pin Lai, Chih-Wei Lu, Weng-Chung Wang, Shih-Chung Chen, Hsien-Yi Wang, Shih-Bin Su

**Affiliations:** 1 Department of Management, Chi-Mei Medical Center, Tainan, Taiwan; 2 Department of Hospital and Health Care Administration, Chia-Nan University of Pharmacy and Science, Tainan, Taiwan; 3 Department of Emergency Medicine, Chi-Mei Medical Center, Tainan, Taiwan; 4 Department of Industrial and Systems Engineering, Chung Yuan Christian University, Chung Li, Taiwan; 5 Assessment Research Center, The Hong Kong Institute of Education, Hong Kong, China; 6 Institute of Biomedical Engineering, Southern Taiwan University, Tainan, Taiwan; 7 Division of Nephrology, Department of Medicine; Chi-Mei Medical Center, Tainan, Taiwan; 8 Department of Sports Management, College of Leisure and Recreation Management, Chia Nan University of Pharmacy and Science, Tainan, Taiwan; 9 Department of Family Medicine, Chi-Mei Medical Center, Tainan, Taiwan

## Abstract

**Background:**

To develop a web-based computer adaptive testing (CAT) application for efficiently collecting data regarding workers' perceptions of job satisfaction, we examined whether a 37-item Job Content Questionnaire (JCQ-37) could evaluate the job satisfaction of individual employees as a single construct.

**Methods:**

The JCQ-37 makes data collection via CAT on the internet easy, viable and fast. A Rasch rating scale model was applied to analyze data from 300 randomly selected hospital employees who participated in job-satisfaction surveys in 2008 and 2009 via non-adaptive and computer-adaptive testing, respectively.

**Results:**

Of the 37 items on the questionnaire, 24 items fit the model fairly well. Person-separation reliability for the 2008 surveys was 0.88. Measures from both years and item-8 job satisfaction for groups were successfully evaluated through item-by-item analyses by using *t*-test. Workers aged 26 - 35 felt that job satisfaction was significantly worse in 2009 than in 2008.

**Conclusions:**

A Web-CAT developed in the present paper was shown to be more efficient than traditional computer-based or pen-and-paper assessments at collecting data regarding workers' perceptions of job content.

## Background

Many previous studies have reported on the relationships between job satisfaction, psychological distress, psychosocial processes and stress-related biological factors [1-5]. Amati et al. [1] reported that job satisfaction is related to psychological stress affecting cellular immune function and that changes in work satisfaction over time could affect the immunological-inflammatory status of workers. Optimizing the ways in which healthcare providers use institutional services to maximize the likelihood of positive health outcomes is thus urgent and essential [6,7].

### 1. Standardized assessments of health status

Within survey or research settings, there are two routinely used forms of standardized health status assessments [8].

(1) A lengthy and structured interview conducted by experts to systematically investigate the presence and nature of each symptom of every disorder (this is often considered the ''gold standard'' in psychiatric diagnosis by researchers [9,10], but it requires significant amounts of time and training to administer).

(2) A rapid assessment instrument that attempts to briefly screen for the most common symptoms of psychiatric disorders by using a cut-off point to identify degrees of impairment based on specific scores (e.g., sleep, the quality-of-life scale[11], the Job Content Questionnaire (JCQ)[12], and the Beck Anxiety and Depression Inventories[13]).

The length and complexity of many fixed-form instruments are problematic and raise concerns about both the burden on respondents and the administration costs [14,15]. Conversely, the shift to shorter fixed-form versions of patient-reported instruments has raised concern over possible resultant losses of precision and reliability [16] as well as insensitivity to clinically meaningful changes [17].

### 2. CAT reduces the burden on patients and diagnosticians

Studies have shown that computer adaptive testing (CAT) can save time and alleviate the burdens on both examinees (e.g., patients) and test administers (e.g., diagnosticians), as compared to traditional computer-based or pen-and-paper assessments [18-21]. CAT, which is based on item response theory (IRT)[21], is a test-administration method that tailors the assessment to the latent-trait level of the examinee. Only items that are neither too hard, nor too easy, are administered. IRT-based CAT has attracted much attention because of its better control of item exposure and lower cost of item development for medical and healthcare professionals [22,23]. CAT can efficiently collect data from examinees and identify the degree of severity of each symptom of disorder. Thus, CAT overcomes the shortcomings of the two traditional forms of standardized assessments in clinical settings, both the burdens associated with lengthy assessments and the loss of precision and reliability of shorter fixed-form assessments.

### 3. Item-by-item questionnaire analyses

Although CAT and the aforementioned lengthy and short assessments are all used to obtain composite scores for measurement, item-by-item analyses are also common in research reports. In item-by-item analyses, perception changes between groups are compared across items. One item (or one composite score) is assessed at a time [22] by traditional one-way ANOVA, by a *t*-test, or even by Pearson's *chi*-square test [6]. Recently, item-by-item skewness analysis by a bootstrapping procedure has been reported as effective for identifying quality-of-life concerns of patients [24]. The problem we face when using CAT is how to obtain the specific responses interacted by item and person because only individual measures were stored in the CAT module.

### 4. Study Objectives

This study aimed to answer two questions: (1) Can a CAT be used via a website to facilitate more efficient response collection for the self-evaluation of job satisfaction by workers? and (2) Is it possible to generate data using the Rasch model (1960) to assess achievement through item-by-item analysis?

## Methods

### 1. Study participants and research instrument

The study was conducted in a 1,200-bed hospital in Taiwan. One-tenth of hospital employees were randomly enrolled for surveys of job satisfaction in September of 2008 and 2009. The self-administered 37-item Job Content Questionnaire (JCQ-37) was designed for use on a website via NAT (non-adaptive testing) in 2008 and CAT assessments with 24 items in 2009 was provided to workers. The response rates were 92.6% and 91.1% for 2008 and 2009, respectively. This study was approved and monitored by the administration units of the hospital.

### 2. Instrument selection

#### (1) Questionnaire

Eight items related to supervisors and coworker-support in the Chinese version of the JCQ (C-JCL) [25] were combined with 29 other items regarding job satisfaction to form the 37-item Job Content Questionnaire (JCQ-37). The questionnaire covered the following six domains: welfare and the environment (measured by eight items), institutional image (measured by five items), intra- and inter-department relationship (measured by seven and five items, respectively) and personal professional learning and working conditions (measured by five and seven items, respectively). For each item, the response was recorded using a four-point Likert scale ranging from 1 (strongly disagree) to 4 (strongly agree).

#### (2) Rasch analysis

We constructed a user-friendly Web-CAT self-rated questionnaire assessment to help provide hospital services based on individual needs as identified from relevant descriptions of job satisfaction. Construction of a unidimensional assessment to measure job satisfaction was required. The Rasch rating scale model [26,27] and WINSTEPS software [28] were used to examine the 2008 responses to JCQ-37 by workers and to determine whether these responses could form a unidimensional measurement. The items meeting the requirements of the Rasch model (unidimensionality and data-model fit) were the items used to construct the Web-CAT in 2009.

#### (3) Unidimensionality

Rasch modeling has been reported to be superior to factor analysis for confirming one factor structure [29]. Using Rasch analyses to assess unidimensionality has been the subject of much discussion in the literature [30-33]. Tennant and Pallant [34] and Richard Smith [35] suggested that exploratory factor analysis (EFA), especially using parallel analysis [36], should be undertaken to assess the dimensionality of the study data. Several studies [24,37-39] have used principal component analysis (PCA) of the standardized residuals to verify that items fit the assumption of unidimensionality. Certain criteria are suggested to determine whether the standardized residuals conform to unidimensionality: 1) a cutoff at 60% of the variance explained by the Rasch factor and 2) the first eigenvalues on residuals smaller than 3 and the percentage of the variance explained by the first contrast of less than 5% [40,41]. Poor-fitting items with a mean square error (MNSQ) beyond the range of 0.5-1.5 were discarded from the questionnaire to guarantee unidimensional interval measures in a logit unit (i.e., log odds) [27,40,42].

### 3. Web-CAT assessment

We designed a CAT questionnaire that complies with rules and criteria for CAT-based testing on the internet http://www.healthup.org.tw/irt_test4/irt_start.htm.

Based on person-separation reliability (e.g., Rasch_*rel*, similar to Cronbach's alpha) calculated from the job-satisfaction survey conducted in 2008, the CAT termination rule for measurement of standardized error (MSE) is determined by formula (1)[43].(1)

where, *SD*_x _represents the standard deviation of person measures estimated in 2008. We also defined another termination rule for CAT so that the minimum number of items required for completion of the CAT questionnaire was 10. The initial item was selected according to the overall job-satisfaction level designated by the examinee's response at the beginning of the CAT questionnaire. When an examinee rated the CAT questionnaire after completing three items on the web, the computer could update the estimate of the examinee's satisfaction level (ability) after each subsequent item's answer was complete. The provisional-person measures was estimated by the iterative Newton-Raphson procedure [18,44], a brief algorism was presented in Additional file 1. The next item selected was that with the most information about the provisional-person measures in the remaining unanswered items.

### 4. Generation of person responses across items

Only individual measures were stored in the CAT module. We should thus generate appropriate responses for each person and each item so that item-by-item comparisons can be made over several years. A standard item-response generation method, as used in previously published papers [24,45-48], was conducted using the Rasch rating scale model. An Excel routine was demonstrated in Additional file 1.

## Results

### 1. Descriptive Statistics

Table 1 compares the demographic characteristics of the study sample in 2008 and 2009. The average age and the mean duration of work tenure were 34 and 8.5 years, respectively. The majority of respondents were female (79%) and only 12-14% were physicians. Chi-square tests showed that gender, occupation, age and work tenure were not significantly different between the two assessment years (*p *> 0.05).

**Table 1:** Comparison of demographic characteristics of the 2008 and 2009 samples

Year	2008(n = 297)		2009(n = 291)		**Prob.**^**a**^
Sample	N	(%)	N	(%)	
**Gender**					
**Female**	236	79.46%	228	78.35%	0.819
**Male**	61	20.54%	63	22.65%	
**Job title**					
**Administrator**	45	15.15%	51	15.84%	0.597
**Nurse**	137	46.13%	144	44.72%	
**Physician**	37	12.46%	47	14.60%	
**Technician**	78	26.26%	87	27.02%	
**Average age (yrs)**					
**18~25**	31	10.44%	29	9.96%	0.299
**26~35**	155	52.19%	150	51.50%	
**36~45**	85	28.62%	80	27.40%	
**46~55**	20	6.73%	29	9.96%	
**≧ 56**	6	2.02%	2	0.68%	
**Work tenure**					
**< 1 yr**	14	4.71%	15	5.15%	0.283
**1-3**	64	21.55%	56	19.20%	
**4-7**	61	20.54%	81	27.80%	
**8-14**	104	35.02%	86	29.50%	
**≧ 15**	54	18.18%	52	17.80%	

Tenure: Mean(SD)	8.84(6.51)		8.38(6.43)		
Age: Mean(SD)	34.22(7.73)		34.23(7.83)		

a: Χ^2 ^test

### 2. Unidimensional validity and the identification of concerns

Of the 37 items, 24 items in the 2008 survey, fit the expectations of the Rasch model well, with an Infit MNSQ range of 0.50-1.50 (shown in Table 2). The most difficult (i.e., rarest in frequency) item to obtain was a well-designed hospital-to-worker message delivery system (item 11; 2.73 logits in 2008). In contrast, the easiest (i.e., most common occurrence) was always maintaining a happy mood at work (item 33; -0.68 logits in 2008). Person-separation reliability was 0.88 for 2008. The standard deviation and mean of person measures were 1.99 and 2.30, respectively. The termination rule for CAT was thus set at SEM = 0.68 [1.99 × sqrt(1-0.88)] according to formula (1).

**Table 2:** Item difficulty in logit, SE, MNSQ of Infit and Outfit surveyed in 2008

1-4 scale hospital-based employee satisfaction questionnaire		Item		MNSQ	
No.	with possible responses of worse, bad, good and excellent ...	Difficulty	SE	Infit	Outfit
1	Working environment and necessary equipment are	misfit			
2	Feelings regarding the office and staff lounge are	misfit			
3	Parking lots and vehicular traffic indications are	misfit			
4	Meals provided to employees by hospital are	misfit			
5	Hospital's disaster prevention ability is	misfit			
6	Overall feeling of the current work environment is	misfit			
7	Hospital benefits and salary provided to employees are	misfit			
8	Salary and wage levels compared with other hospitals are	0.47	0.17	1.12	1.14
9	The performance appraisal system is open, fair and reasonable	misfit			
10	My objective is closely consistent with hospital goals	0.5	0.17	1.15	1.09
11	The hospital message delivery design works well for workers	2.73	0.13	1.12	0.97
12	The Plan-Do-Check-Action and review measures are	-0.96	0.18	1.03	1.03
13	The hospital work environment compared to others is	0.02	0.18	0.68	0.44
14	Colleagues can cooperate with each other to achieve goals	1.18	0.16	0.93	0.95
15	Interpersonal relationships with colleagues are harmonious	1.75	0.14	0.9	1
16	My boss can give clear instructions to designate tasks	1.03	0.16	0.93	0.91
17	My boss often makes appropriate decisions	-0.46	0.18	1.08	1.13
18	My boss fully shoulders and assumes accountability	-0.11	0.18	1.11	1.22
19	Communication and interaction with my boss is	-0.37	0.18	0.71	0.56
20	Overall, my boss performance can be scored as	-0.87	0.18	0.95	0.91
21	Opportunities to exchange and share experiences with colleagues are	misfit			
22	Opportunities to cooperate and communicate with other departments are	misfit			
23	I often work together with colleagues to achieve objectives	-1.03	0.17	1.11	1.16
24	Harmonious relations with members of other departments are	-0.46	0.18	0.86	0.83
25	Opportunities to interact with members of other departments are	-0.49	0.18	1.05	0.97
26	I can fully extend my professional competence and talent	-0.65	0.18	0.82	0.78
27	Many learning and growth opportunities are available for me	-0.59	0.18	0.97	0.96
28	My job is challenging	-0.24	0.18	0.85	0.84
29	My job provides a sense of identity and accomplishment	0.17	0.18	1.25	1.4
30	My hospital provides necessary on-the-job training courses	0.23	0.17	1.09	1.05
31	Workload and working hours are well allocated	-0.14	0.18	0.99	1.01
32	I can engage in my work, career planning and future vision	-0.52	0.18	0.95	1
33	I am always able to maintain a happy mood at work	-0.68	0.18	1.08	1.04
34	My job burdens do not interfere with my family life	-0.49	0.18	0.94	0.86
35	I can afford my living expenses with income from my job	misfit			
36	This satisfaction survey can be expected to improve the workplace	misfit			
37	I would recommend the hospital if my relatives needed treatment	misfit			

Note. Step-threshold difficulties under the Rasch-rating scale model were -4.16, -1.50 and 2.66.

The principal components analysis of the residuals demonstrated that the 24-item scale accounted for 52.2% of the raw variance explained by the measures. The first contrast had an eigenvalue of 1.8 (less than 3 [41]) and accounted for 4.2% (less than 5% [40]) of the total variance, suggesting that the 24-item scale can be regarded as substantially unidimensional. A parallel analysis also indicated that the 24-item questionnaire regarding job satisfaction measures a common entity. These findings indicate that these 24 items measured a single construct for job satisfaction. The three intersection parameters (also called the step calibrations [48]) under the Rasch rating scale model for the 24-item questionnaire were set at -4.16, -1.50 and 2.66 logits. These thresholds are congruent with the guidelines proposed by Linacre [49] as follows: (1) average measures advance monotonically within each category, (2) step calibrations advance, (3) step difficulties advance by at least 1.4 logits and (4) step difficulties advance by less than 5.0 logits.

### 3. Web-CAT performance

Based on the finding of a unidimensional construct in Table 2, we embedded the stop rules of SEM = 0.68 and the minimal corresponding item length = 10 into the CAT questionnaire. The Web-CAT is at http://www.healthup.org.tw/irt_test4/irt_start.htm.

Table 3 shows an example of a CAT report: (1) The person measure (θ) begins to be estimated at step 4. The final logit is -1.08 and is stopped at step 10 when SE is equal to or less than a SEM of 0.68. (2) The probabilities corresponding to each item difficulty (δ) are in agreement with formula (2) under the Rasch rating scale model [26]:(2)

**Table 3:** Web-CAT for item-selection and response-history reports

Step	Item difficulty	Prob.	Your response	Expected score	Estimated ability	Standard error	Outfit MNSQ
1	0.50	0.17	0	0.80	0.50	-	-
2	1.75	0.06	0	0.32	0.50	-	-
3	-0.46	0.35	1	1.57	0.50	-	-
4	2.73	0.02	2*	0.26	0.50	0.73	2.12
5	-1.03	0.49	0*	1.84	-5.48	0.65	1.71
6	1.18	0.09	1	0.44	-1.55	0.62	2.88
7	1.03	0.11	1	0.49	-0.33	0.57	2.25
8	-0.96	0.47	2	1.82	0.35	0.54	2.73
9	-0.87	0.45	2	1.79	0.41	0.51	2.33
10	-0.68	0.40	2	1.70	-1.08	0.48	1.82

**Figure 1:**
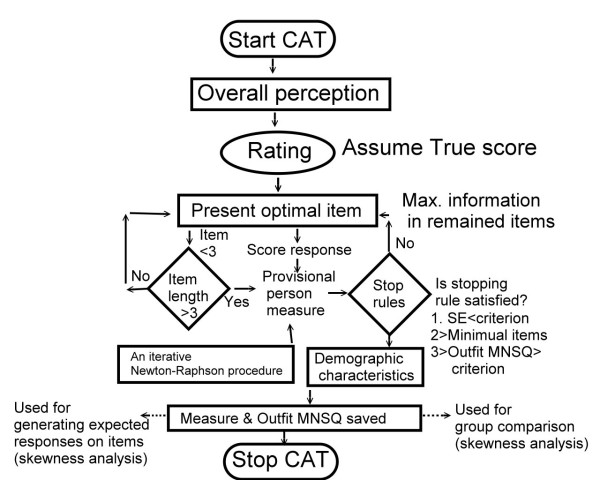
**Procedure and flowchart of CAT**.

A * indicates a significantly unexpected response (*p *< .05). In reference to the expected score in column 5, these should be responded to with 2 and 0 in steps 4 and 5 according to the job satisfaction level of -1.08 logits in step 10. Website available at: http://www.healthup.org.tw/irt_test4/irt_start.htm

where *P_nij _*and *P_ni(*j-*1) _*are the probabilities of being scoring *j *and *j *- 1 in item *i *for person *n*, θ*_n _*is the ability of person *n*, δ*_i _*is the difficulty of item *i*, and τ*_j _*is the *j*-th step difficulty. (see Additional file 1). (3) Outfit MNSQ for CAT was determined by the average squared residuals (i.e., squared observation minus the expected score and then divided by the variance, see Additional file 1) across all items. The outfit MNSQ terminated the CAT procedures once the item length was longer than 10 or the MNSQ was greater than 10. An outfit MNSQ of greater than 2.0 was referred to the aberrant responses given by the person [50](Figure 1). We assumed that aberrant respondents, participants' guessing, inattentiveness, carelessness and coaxing could be caused by fatigue, misunderstanding, or a poor fit of the examinee for evaluation based on item-response theory [51,52]. Z-scores beyond +/- 1.96 were marked on observation with a symbol * to designate that an unexpected response was given to a specified item (*p *< .05).

### 4. Item difference between years

Taking item 8 (salary and wage levels compared with other hospitals) as an example, we examined differences between 2008 and 2009 with the *t*-test, shown in Table 4. In general, the 2008 perceptions had a higher mean score (i.e., more satisfied) than those in 2009, except that the participants aged greater than 55 showed no difference on item 8 between years. Other items were analyzed similarly. Due to space constraints, the results are not reported but available on request.

**Table 4:** Comparison of job perception on item 8 for demographic variables using the *t*-test

Demographic	Difference		95% CI		Test *t*	
Variable	Mean	SE	Upper	Lower	*t*-value	Sig. (2-tailed)
Year	0.82	0.04	0.74	0.91	19.72	<.0001
Gender						
Male	0.76	0.10	0.00	0.76	7.74	<.0001
Female	0.84	0.05	0.75	0.93	18.33	<.0001
Job title						
Administrator	0.85	0.09	0.67	1.03	9.40	<.0001
Nurse	0.82	0.06	0.70	0.94	13.51	<.0001
Physician	0.96	0.09	0.79	1.13	11.18	<.0001
Technician	0.72	0.12	0.48	0.96	6.02	<.0001
Average age (yrs)						
18~25	0.90	0.13	0.63	1.16	6.73	<.0001
26~35	0.81	0.05	0.70	0.91	14.83	<.0001
36~45	0.78	0.08	0.61	0.94	9.29	<.0001
46~55	0.93	0.15	0.63	1.23	6.27	<.0001
≧ 56	1.50	0.50	-0.65	3.65	3.00	0.095
Work tenure						
< 1 yr	0.93	0.18	0.57	1.30	5.24	<.0001
1-3	0.80	0.10	0.61	0.99	8.44	<.0001
4-7	0.70	0.08	0.55	0.85	9.26	<.0001
8-14	0.86	0.08	0.70	1.02	10.44	<.0001
≧ 15	0.94	0.09	0.77	1.12	10.65	<.0001

## Discussion

### 1. Features

#### (1) Key findings

The very group worthy of concern for the studied hospital is workers aged 26-35, who had a substantially lower job satisfaction in 2009 than in 2008. Female nurses with work tenure beyond 18 years showed the most significant deterioration, whereas workers aged greater than 55 showed no difference, on item 8 (salary and wage levels compared with other hospitals) between 2008 and 2009.

#### (2) What this study contributes to current knowledge

This study develops a CAT to examine workers' perceptions of job satisfaction and demonstrates its advantages in reducing the burdens associated with lengthy assessments and improving the measurement precision than non-adaptive testing.

#### (3) Implications of the results and suggested actions

There were two major implications: (1) The Web-CAT (especially when adopting a polytomous as opposed to a dichotomous item design) can be used as a tool for hospital workers to measure their perceptions of job satisfaction, and (2) a standard item-response generation method referring to individual measures estimated by CAT could be applied to item-by-item comparisons. An Excel routine was demonstrated in Additional file 1.

### 2. Study strengths

#### (1) Using CAT and the t-test to compare individual differences on measures and items across years

From a management perspective, promotion of the health of workers has emerged as an important issue [53,54]. Many workplaces now routinely conduct job-satisfaction surveys for employees. Using a questionnaire to measure differences between groups and across items over several years is thus necessary. Providers can rapidly obtain input from workers by means of the results of Web-CAT assessments for individual examinees and the t-test for specific items (or composite scores). Such evaluation is useful for individual and group comparison.

#### (2) Web-CAT saves time and reduces burdens compared with traditional non-adaptive tests

To maximize the likelihood of achieving a desired health promotion outcome, workers are provided with a Web-CAT report that reveals their perceptions of job satisfaction. In contrast to traditional non-adaptive assessment methods, this feature saves time and alleviates burdens on examinees and diagnosticians by immediately transmitting messages. The system also can detect aberrant responses with CAT report cards (Table 3), by outfit MNSQ [47] and by Z-residual scores [18,22,24,27]. By identifying unexpected responses to items, diagnosticians are more likely to notice when feedback messages contain unexpected responses from individual examinees.

#### (3) Polytomous CAT module developed in this study

Many studies investigating IRT- and CAT-based tests using dichotomous items have evaluated both the efficiency and precision of CAT-based tests in the educational, psychometrical and medical fields. However, few studies examine CAT with polytomous items applied to satisfaction surveys. This study especially demonstrated a Web-CAT module for interested readers to practice at http://www.healthup.org.tw/irt_test4/irt_start.htm.

### 3. Study limitations

Because many studies have shown that CAT can save time and alleviate burdens on examinees compared to traditional non-adaptive computer-based or pen-and-paper assessments [18-21], we thus did not demonstrate the efficiency and precision of CAT as compared to non-adaptive assessments. Obtaining high quality examinee feedback from CAT assessments is essential to produce accurate results, and adequate training is required to facilitate an efficient health-promotion system. Without such results and training, it will be extremely difficult for readers to understand the computation of outfit and infit statistics with regard to probability and outfit MNSQ disclosed in Table 3. In this study, the job-satisfaction questionnaire was used as a tool to collect information about workers' perceptions using the CAT feedback system. Accordingly, diagnosticians may need training to interpret the results of the data adequately.

### 4. Problems in application and daily use

#### (1) Applications of CAT

Traditionally, all examinees' responses have to be collected and saved for further analyses, which can be very tedious. In this study, we used the Web-Cat at http://www.healthup.org.tw/irt_test4/irt_start.htm to record item responses of all examines. One can easily apply CAT to any kind of questionnaires. The availability and accessibility of information technology and item response theory makes CAT implementation simple and easy. Those who are interested in CAT implementation can consult the textbook [42] and the following websites: http://www.eddata.com/resources/publications/EDS_Rasch_Demo.xls (for information on the iteration of person estimation and item calibration), http://www.rasch.org/rmt/rmt34e.htm (for information on the computation of outfit and infit statistics) and http://www.rasch.org/rmt/rmt213a.htm (for information on the method to simulate Rasch data). Other relevant information regarding CAT algorithms such as the Newton-Raphson method, item information and SE are shown in Additional file 1.

#### (2) Generation of person responses across items

It is impossible to collect all the necessary response data as traditional computer-based or pen-and-paper assessments when applying CAT. Person responses across all items should be statistically yielded if item-by-item analyses across groups are required for comparisons. The standard item-response generation method introduced in previously published papers [24,45-48] is worth consulting for further reference.

## Conclusion

The outcomes of this study, especially for the item parameters presented in Table 2, imply that the Web-CAT is a useful tool for examining job satisfaction in hospital work sites. Future studies can further investigate the job-satisfaction cut-off point for hospital workers for the purpose of improving job-satisfaction perceptions and promoting mental health in the workplace. A Web-CAT with graphs and animations will be developed by the authors in the near future.

## List of abbreviations

CAT: computer adaptive testing; EFA: exploratory factor analysis; JCQ: job content questionnaire; IRT: item response theory; MNSQ: mean square error; MSE: standardized error of measurement; NAT: non-adaptive testing; PA: parallel analysis; VBA: visual basic for application

## Competing interests

The authors declare that they have no competing interests.

## Authors' contributions

TW and SB provided the concepts and ideas for the research design, writing, data analysis, facilities and equipment and fund procurement. WP and WC provided the institutional liaison and project management. CW, SC, HY and WP provided consultation (including English revision and review of the manuscript before submission).

## Pre-publication history

The pre-publication history for this paper can be accessed here:

http://www.biomedcentral.com/1471-2288/11/47/prepub

## Supplementary Material

Additional file 1 **Expected scores obtained by the Rasch model's probability theory**. Excel-VBA program for randomly generating Rasch model's expected scores. Click here for file
